# Aortic carboxypeptidase-like protein enhances adipose tissue stromal progenitor differentiation into myofibroblasts and is upregulated in fibrotic white adipose tissue

**DOI:** 10.1371/journal.pone.0197777

**Published:** 2018-05-25

**Authors:** Mike Jager, Mi-Jeong Lee, Chendi Li, Stephen R. Farmer, Susan K. Fried, Matthew D. Layne

**Affiliations:** 1 Department of Biochemistry, Boston University School of Medicine, Boston, Massachusetts, United States of America; 2 Section of Endocrinology, Diabetes, and Nutrition, Department of Medicine, Boston University School of Medicine, Boston, Massachusetts, United States of America; University of Rochester School of Medicine and Dentistry, UNITED STATES

## Abstract

White adipose tissue expands through both adipocyte hypertrophy and hyperplasia and it is hypothesized that fibrosis or excess accumulation of extracellular matrix within adipose tissue may limit tissue expansion contributing to metabolic dysfunction. The pathways that control adipose tissue remodeling are only partially understood, however it is likely that adipose tissue stromal and perivascular progenitors participate in fibrotic remodeling and also serve as adipocyte progenitors. The goal of this study was to investigate the role of the secreted extracellular matrix protein aortic carboxypeptidase-like protein (ACLP) on adipose progenitor differentiation in the context of adipose tissue fibrosis. Treatment of 10T1/2 mouse cells with recombinant ACLP suppressed adipogenesis and enhanced myofibroblast differentiation, which was dependent on transforming growth factor-β receptor kinase activity. Mice fed a chronic high fat diet exhibited white adipose tissue fibrosis with elevated ACLP expression and cellular fractionation of these depots revealed that ACLP was co-expressed with collagens primarily in the inflammatory cell depleted stromal-vascular fraction (SVF). SVF cells isolated from mice fed a high fat diet secreted increased amounts of ACLP compared to low fat diet control SVF. These cells also exhibited reduced adipogenic differentiation capacity in vitro. Importantly, differentiation studies in primary human adipose stromal cells revealed that mature adipocytes do not express ACLP and exogenous ACLP administration blunted their differentiation potential while upregulating myofibroblastic markers. Collectively, these studies identify ACLP as a stromal derived mediator of adipose progenitor differentiation that may limit adipocyte expansion during white adipose tissue fibrosis.

## Introduction

In response to chronic caloric excess, white adipose tissue (WAT) exhibits increased inflammation [1,2] increased hypoxia [3] and fibrotic remodeling [4,5]. WAT fibrosis is recognized to be a major contributor of metabolic dysfunction [6–8] and hypothesized to limit WAT hyperplasia by blunting the differentiation of progenitors into adipocytes [9–11]. In other fibrotic tissues, myofibroblasts are a critical cell type which are characterized by elevated α-smooth muscle actin (SMA) expression and extracellular matrix (ECM) protein production, including collagen 1 (Col1) [12]. Myofibroblasts drive fibrosis via both ECM secretion and contractile remodeling resulting in stiff fibrous scars [12]. While several cell types likely contribute to WAT fibrosis, including adipocytes [7] and macrophages [11,13,14], other studies have highlighted the contribution of progenitor differentiation pathways and ECM remodeling in fibrosis [9,15].

Several effectors regulate WAT fibrosis including transforming growth factor-β (TGFβ), a pro-fibrotic [16,17] and anti-adipogenic [18] cytokine, that is increased with obesity [19] and directs myofibroblast differentiation in adipose progenitors [11]. WAT fibrosis is characterized by the accumulation of several collagens including types I, III, and VI [6]. The importance of specific collagens in WAT fibrosis is supported by studies showing that a cleavage product of Col6a3, endotrophin, regulates fibrosis [20] and genetic ablation of collagen VI protects mice from metabolic disorders [7].

Aortic carboxypeptidase-like protein (ACLP), gene name adipocyte enhancer binding protein 1 (*AEBP1*) [21,22], is a secreted ECM-associated protein expressed primarily by perivascular and vascular cells which is upregulated in activated vascular cells following vascular injury [23]. ACLP is composed of an N-terminal signal sequence, a charged lysine, proline, and glutamic acid-rich domain, a collagen binding discoidin domain and a catalytically inactive metallocarboxypeptidase domain [24–26]. The mouse Aebp1 protein was originally described as a transcriptional repressor and regulator of energy metabolism in WAT [21,27], however it is now generally recognized that Aebp1 is a partial clone of ACLP [28]. Several studies have demonstrated that the vascular niche is a source of adipogenic precursors [29–33]. In addition, our previous work has established that ACLP is expressed in vascular smooth muscle cells and also in the vasculature in both subcutaneous and visceral adipose depots [23,34,35]. While others have observed that ACLP expression is negatively regulated during adipogenesis [21,22,36,37], the function of ACLP in adipocyte progenitor differentiation and WAT fibrosis remains largely unknown.

The goal of this study was to examine the expression of ACLP in fibrotic WAT and to investigate its role in regulating the differentiation of adipose progenitors. In vitro studies in mouse 10T1/2 cells determined that ACLP repressed adipogenesis and enhanced myofibroblast differentiation through TGFβ receptor (TGFβR) signaling. Histological analysis and tissue fractionation of high fat diet (HFD) induced fibrotic WAT determined that the stromal-vascular fraction (SVF) exhibited increased ACLP protein expression and ACLP was co-expressed with collagens. Additionally, inflammatory cell depleted SVF populations were the primary source of ACLP expression in fibrotic WAT. Examination of human adipose stromal cells (hASC), determined that ACLP repressed their differentiation into mature adipocytes and enhanced their differentiation into myofibroblasts. Collectively, these studies identify ACLP as a stromal derived positive mediator of myofibroblast differentiation in adipose tissue progenitors that accumulates in fibrotic adipose tissue.

## Materials and methods

### Cell culture

De-identified human adipose stromal cells (hASC) were obtained from the Boston Nutrition Obesity Research Center Adipocyte Biology Core, with approval by the Institutional Review Board of Boston University Medical Center. All subjects provided informed consent. C3H/10T1/2 (10T1/2) (ATCC) fibroblasts and hASC were cultured in 1:1 Ham’s F12:DMEM supplemented with 10% fetal bovine serum (FBS, Atlas Biochemicals) and 1% penicillin/streptomycin and incubated in a 5% CO_2_ atmosphere at 37°C. Confluent 10T1/2 fibroblasts were differentiated into adipocytes with 5 μM dexamethasone, 500 μM 3-isobutyl-1-methylxanthine (IBMX), 860 nM insulin, and 125 μM indomethacin (DMII). After 2 days, cells were maintained in 10% FBS, 430 nM insulin, for the remainder of the study. Adipocyte differentiation assays of hASC were performed as previously described [38]. Briefly, hASC were grown for 2 days after reaching confluence and then moved to complete differentiation media (CDM) containing 500 μM IBMX, 100 nM insulin, 100 nM dexamethasone, 2 nM triiodothyronine, 10 μg/ml transferrin, 1 μM rosiglitazone, 33 μM biotin and 17 μM pantothenic acid. hASC was in CDM for 7 days total with a replacement on day 4. Following 7 days of CDM, hASC were maintained in serum free media with 10 nM insulin and 10 nM dexamethasone. rACLP was produced and purified as previously described [26]. For analysis of hASC, pre-confluent (80–90% confluent) hASC were treated with 3.75 μg/ml rACLP (~30 nM) everyday for 2 days prior to and on the day of providing CDM. For analysis of 10T1/2 fibroblasts, pre-confluent (80–90% confluent) cells were treated with 3.75 μg/ml rACLP (~30 nM) for 2 days prior to DMII and maintained in the treatment throughout differentiation. Fractionation of day +8 10T1/2 fibroblasts was performed by incubating cells for 5 minutes in 0.05% trypsin-EDTA, re-suspension in 1.02 mg/ml Ficoll-PBS buffer and subsequent centrifugation for 10 min at 400 x g.

### Oil Red-O staining and quantification

To measure lipid accumulation, cells were washed with PBS, fixed with 10% paraformaldehyde for 30 minutes, stained with Oil Red O for 60 minutes followed by washing with PBS. Brightfield images were taken with identical exposures on an Olympus IX70 microscope. Oil Red O was extracted with 100% isopropanol and absorbance was measured at 500 nm using a BioTek Synergy HT plate reader. Empty wells stained with Oil Red O were used as background and absorbance was subtracted from each sample for quantification.

### SDS-PAGE and Western blotting

Total protein lysates were harvested in Western extraction buffer (25 mM Tris pH 7.4, 50 mM sodium chloride, 0.5% sodium deoxycholate, 2% NP-40 and 0.2% sodium dodecyl sulfate) with protease and phosphatase inhibitors (Roche) as described [24]. Protein concentrations were determined with BCA protein assay kit (Thermo Scientific) and equal amounts of protein were run on 4–12% NuPage bis-tris protein gels (Life Technologies), and transferred onto Immobilon-FL PVDF membranes (EMD Millipore) according to standard procedures. Antibodies used include ACLP [35] (1:4000); α-SMA (Sigma A2547 Lot# 084M4795V, 1:4000); collagen α1 (Rockland 600-401-103-0.1 Lot# 33743, 1:1000); FABP4 (Cell Signaling Technology 3544S Lot# 2, 1:2000); cyclophilin A (CypA) (EMD Millipore 07–313 Lot# 2572047, 1:2000); adiponectin (Thermo Scientific PA1054 Lot# PF194392, 1:2000); perilipin (Cell Signaling Technology 9349S Lot# 4, 1:2000); PPARγ (Santa Cruz Biotechnology SC-7196 Lot# F2614, 1:1000); CRP2 [39] (1:1500); Desmin (Dako M0724 Lot# (074)011, 1:500); SM22 (Abcam ab14106 Lot# ab14106, 1:1000). Signal was detected with horseradish peroxidase conjugated secondary antibodies and using ECL substrate (Thermo Scientific) using a Bio-Rad Chemidoc imaging system. For conditioned media samples, equal loading was confirmed by membrane staining with Ponceau S (Sigma). Protein quantification relative to CypA was measured by densitometry using Image Lab software (Bio-Rad).

### Analysis of fibrotic adipose tissue

Male mice fed low fat diet (LFD) (Jackson, D12450B, 10 kcal% fat) or high fat diet (HFD) (Jackson, D12492, 60 kcal% fat) starting at 6 weeks of age until 22 weeks were acquired from Jackson laboratories. Adipose tissues were isolated from 7 LFD and 7 HFD fed male mice and 4 animals were examined by tissue fractionation and 3 each were used for histological analysis. The Boston University School of Medicine Institutional Animal Care and Use Committee approved all animal experiments. For Western blot analysis, epididymal adipose tissue was removed, rinsed 3 times in PBS and finely minced. Minced tissue was digested with 1x dispase (BD Falcon), 1 mg/ml type 1 filtered collagenase (Worthington), 4.5 μg/ml DNase (Worthington) and 1% BSA (Fisher BioReagents) in DMEM for 45 minutes at 37°C. Cells were passed through 100 μm cell strainer and were isolated into floating (adipocyte) and pellet (SVF) fractions using a 1.02 mg/ml Ficoll-PBS buffer by centrifugation. Magnetic-activated cell sorting (MACS) (Miltenyi Biotec) was then performed on the SVF according to manufacturer’s instructions. Briefly, SVF were incubated with anti-CD45 conjugated microbeads. SVF were then passed over a MACS column in a magnetic field and subsequently washed. Flow-through cells were collected as CD45- SVF. The MACS column was then removed from the magnetic field and CD45+ cells were eluted and collected.

### Tissue preparation for histology

Epididymal adipose tissue was rinsed 3 times in PBS, fixed with methyl Carnoy’s fixative (60% methanol, 30% chloroform, 10% glacial acetic acid) for 3 hours at 4°C and then 70% ethanol overnight at 4°C. Tissues were processed, embedded in paraffin and sectioned using standard techniques [34].

### Picrosirius red staining

Sections of the epididymal adipose tissue (10 μm) were deparaffinized, rehydrated, stained with picrosirius red solution (Electron Microscopy Sciences) for 90 minutes at room temperature, rinsed twice with 0.01 N HCl and then dehydrated with increasing concentrations of ethanol and finished in xylenes prior to mounting. Brightfield and polarized light images were obtained using an Olympus IX70 inverted microscope with an Optronics camera.

### Immunofluorescence

Sections of epididymal adipose tissue (10 μm) were rehydrated, blocked with normal goat serum, incubated with ACLP (Thermo Fisher) PA5-23607 Lot# QF2039189, 1:100) or perilipin (Cell Signaling Technology 9349S Lot# 4, 1:200), followed by Alexa Fluor 647 conjugated secondary antibodies (Invitrogen, 1:300). Stained tissues were counterstained with DAPI and mounted. Tissues were also stained with Alexa Fluor 647 conjugated secondary antibody without primary antibody to determine background of immunofluorescence (data not shown). Images were taken at equivalent exposures with a Zeiss Observer D1 equipped with an ORCA-Flash 4.0 digital CMOS camera.

### Quantitative real-time PCR

RNA was isolated from cells using GeneJET RNA purification kit (Therm Fisher) according to the instructions of the manufacturer. cDNA was generated from 200 ng of mRNA using a Maxima First Strand cDNA synthesis kit (Thermo Fisher). Analysis of gene expression was performed using Luminaris Color HiGreen qPCR Master Mix (Thermo Fisher) in an ABI Prism 7300 sequence detector. Intron-spanning primers were used for qPCR reactions. The relative amount of mRNAs was determined through the comparative threshold cycle (ΔΔC_T_) method. PPIA was used as invariant control. Primer sequences are listed in S1 Table.

### Statistical analysis

Data are presented as mean ± S.D. One-way ANOVA with post hoc Tukey’s test were used to compare data between two conditions among multiple conditions. For analysis comparing two conditions where each control replicate was set to 1, a one sample *t* test was used to determine statistical significance. For all other analysis, a Student’s *t* test was used to determine statistical significance. Differences were considered significant when *p* < 0.05.

## Results

### ACLP is predominately expressed in non-differentiated 10T1/2 fibroblasts with limited expression in differentiated 10T1/2 adipocytes

**Fig 1:**
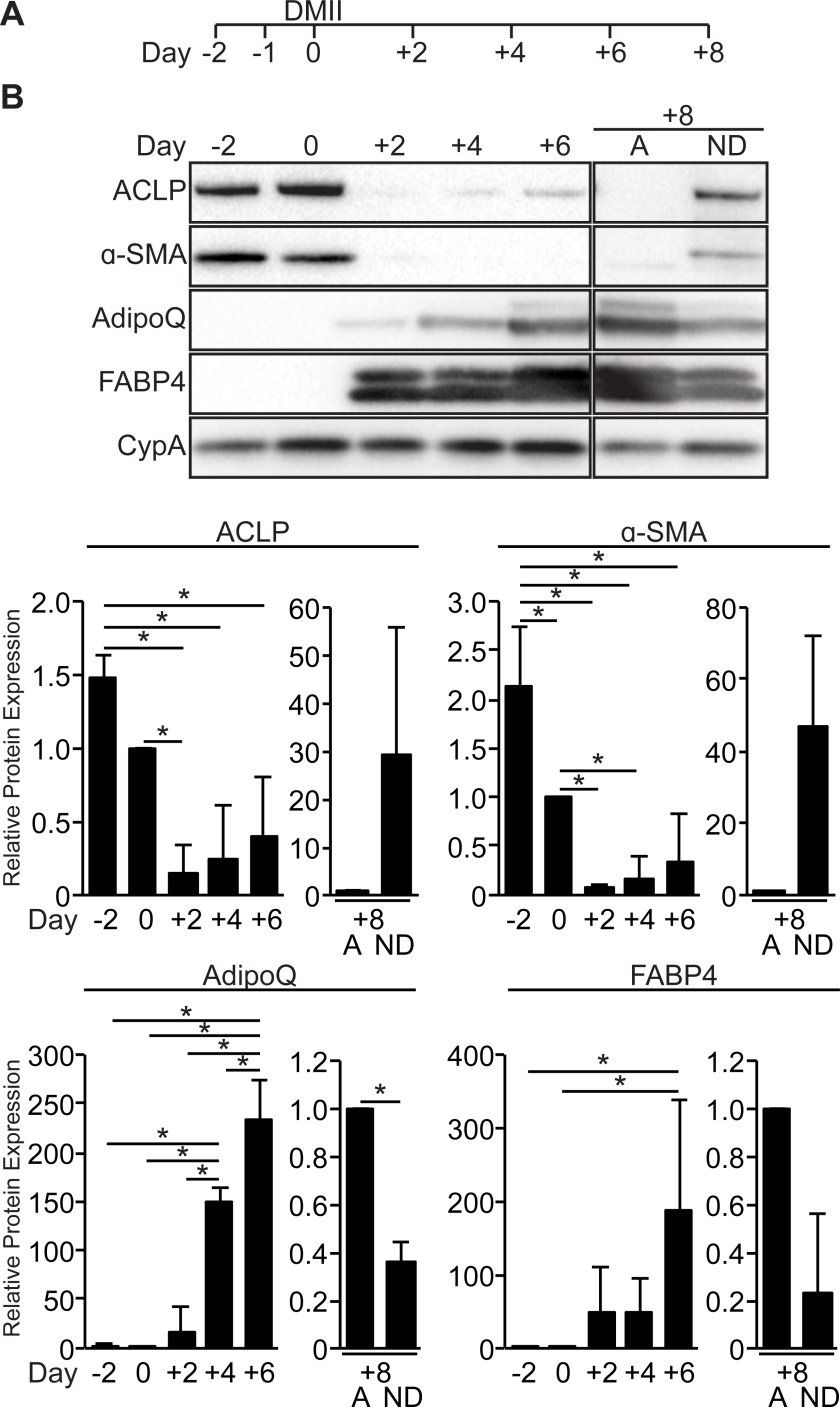
ACLP expression is repressed during adipogenesis. **A**, Scheme of time course for 10T1/2 adipogenic differentiation. **B**, 10T1/2 fibroblasts were induced to undergo adipogenesis with DMII on day 0. Protein was harvested on days -2, 0, +2, +4 and +6. Day +8 cells were fractionated into differentiated adipocytes (A) and the non-differentiated 10T1/2 fibroblasts (ND) and harvested for protein. Protein expression was analyzed using SDS-PAGE and Western blot with antibodies against ACLP, α-SMA, FABP4, adiponectin (AdipoQ) and cyclophilin A. Protein expression was quantified by densitometry normalized to CypA expression and all samples were compared to day 0 cells or mature adipocyte fraction. *, *p* < 0.05 versus control, one way ANOVA with post hoc Tukey’s test for day -2, 0, +2, +4 and +6 samples and one sample t-test for fractionated day +8 samples.

Previous studies have documented the kinetics of ACLP expression during adipogenesis [21,28,36,40], however it is unclear why ACLP expression re-emerges at later time points during adipogenesis. We hypothesized the non-differentiated cells re-expressed ACLP while mature adipocytes no longer expressed ACLP. We differentiated 10T1/2 fibroblasts into mature adipocytes (Fig 1A). 10T1/2 fibroblasts expressed α-SMA and ACLP prior to adipogenic induction and these proteins were substantially diminished with adipogenic induction on day 2 (7% and 14% of day 0 respectively) (Fig 1B). As anticipated, the expression of both adiponectin and FABP4 increased with adipogenic differentiation (Fig 1B). Notably, ACLP expression increased on day 4 and continued throughout terminal differentiation (Fig 1B). To examine if this expression was in the mature adipocytes or in the residual undifferentiated 10T1/2 fibroblasts, the day 8 cells were fractionated by centrifugation based on buoyancy. Mature adipocytes (A) expressed the adipocyte markers, FABP4 and adiponectin, with limited expression of α-SMA and ACLP (Fig 1B). Non-differentiated 10T1/2 fibroblasts (ND) expressed ACLP and α-SMA, with decreased amounts of FABP4 and adiponectin relative to adipocytes. These findings demonstrate an inverse relationship between ACLP, the established progenitor marker α-SMA [29,32], and adipogenesis.

### Recombinant ACLP represses adipogenesis and enhances myofibroblast differentiation in progenitors

**Fig 2:**
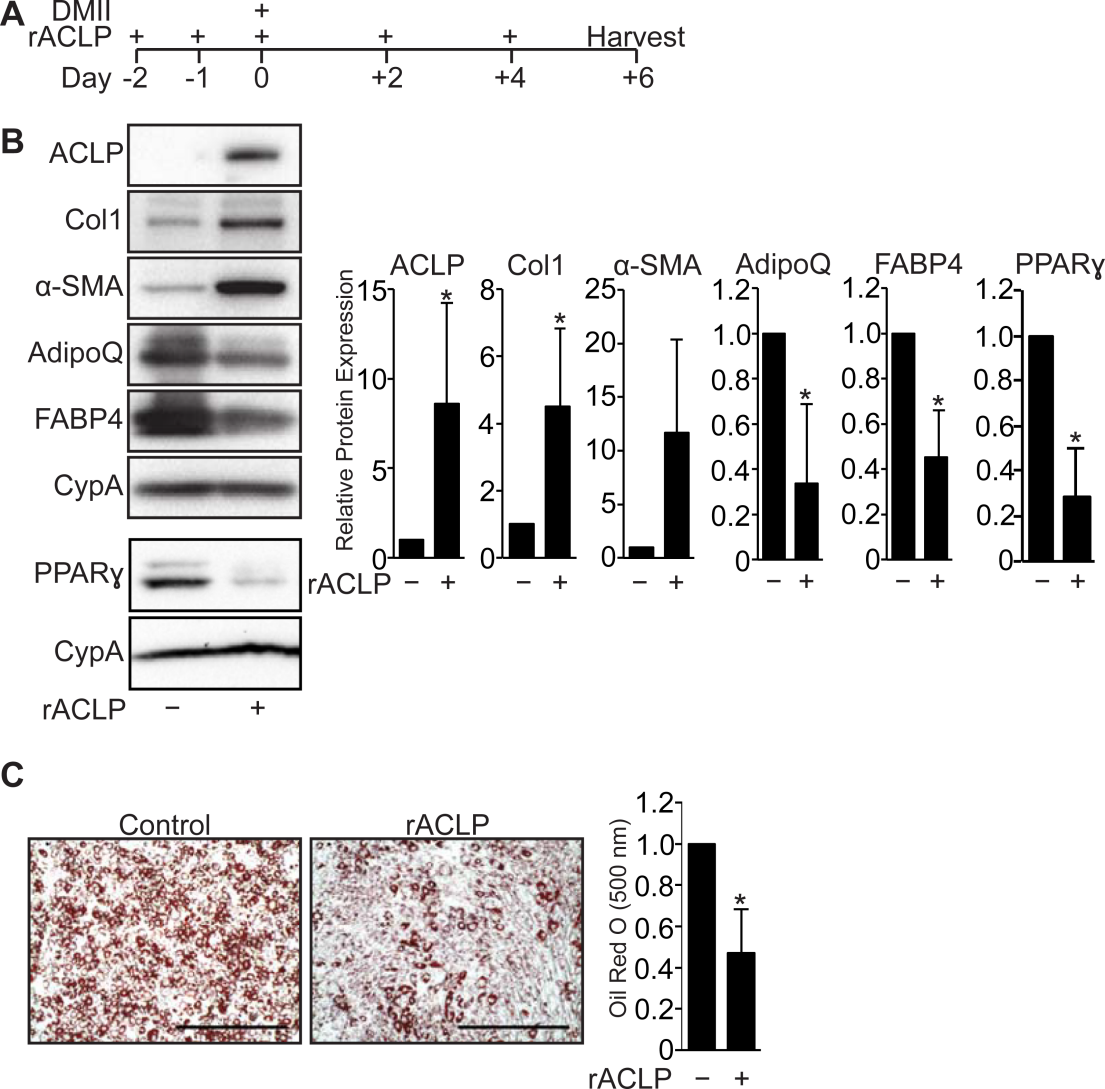
ACLP signaling represses adipogenesis. **A**, Scheme of 10T1/2 adipogenesis and rACLP treatment. **B**, 10T1/2 fibroblasts were treated with 30 nM rACLP on days -2, -1, 0, +2 and +4 and induced to undergo adipogenesis with DMII on day 0. Protein was harvested on day +6 and analyzed by SDS-PAGE and Western blot with antibodies against ACLP, collagen, α-SMA, AdipoQ, FABP4, PPARγ and cyclophilin-a (CypA). PPARγ analysis was performed in separate experiments. Protein expression was quantified by densitometry normalized to CypA expression and relatively compared to control cells. *, *p* < 0.05 versus control, one sample t-test for all values. **C**, 10T1/2 fibroblasts were treated with 30 nM rACLP on days -2, -1, 0, +2 and +4 and induced to undergo adipogenesis with DMII on day 0. On day +6 cells were fixed, stained, imaged and quantified with Oil Red O dye (n = 3). Data were normalized relative to untreated controls. *, *p* < 0.05 versus paired control, one sample t-test. Data presented are expressed as mean ± SD. The scale bar represents 2 mm.

Because ACLP is rapidly down regulated with adipogenesis (Fig 1B) and is a secreted ECM-associated protein [23] that contributes to fibrosis in other organs [41], we next investigated if ACLP blunted adipogenic differentiation by stimulating fibrotic pathways. We treated 10T1/2 cells with recombinant ACLP (rACLP) prior to and following adipogenic induction (Fig 2A). Compared with untreated controls, rACLP treatment significantly inhibited adipocyte differentiation, indicated by lower expression levels of adiponectin, FABP4 and PPARγ (2.7, 1.8, 3.5 fold respectively) (Fig 2B). Additionally, compared with untreated controls, rACLP enhanced myofibroblast differentiation, as indicated by increased ACLP and collagen I (Col I) (8.6 and 4.5 fold respectively) (Fig 2B). rACLP treated cells also contained 55% less lipid measured by Oil Red O accumulation (Fig 2C). These findings demonstrate ACLP shifts progenitor differentiation towards myofibroblast differentiation.

### ACLP-mediated repression of adipogenesis and enhancement of myofibroblast differentiation is dependent on TGFβR signaling

**Fig 3:**
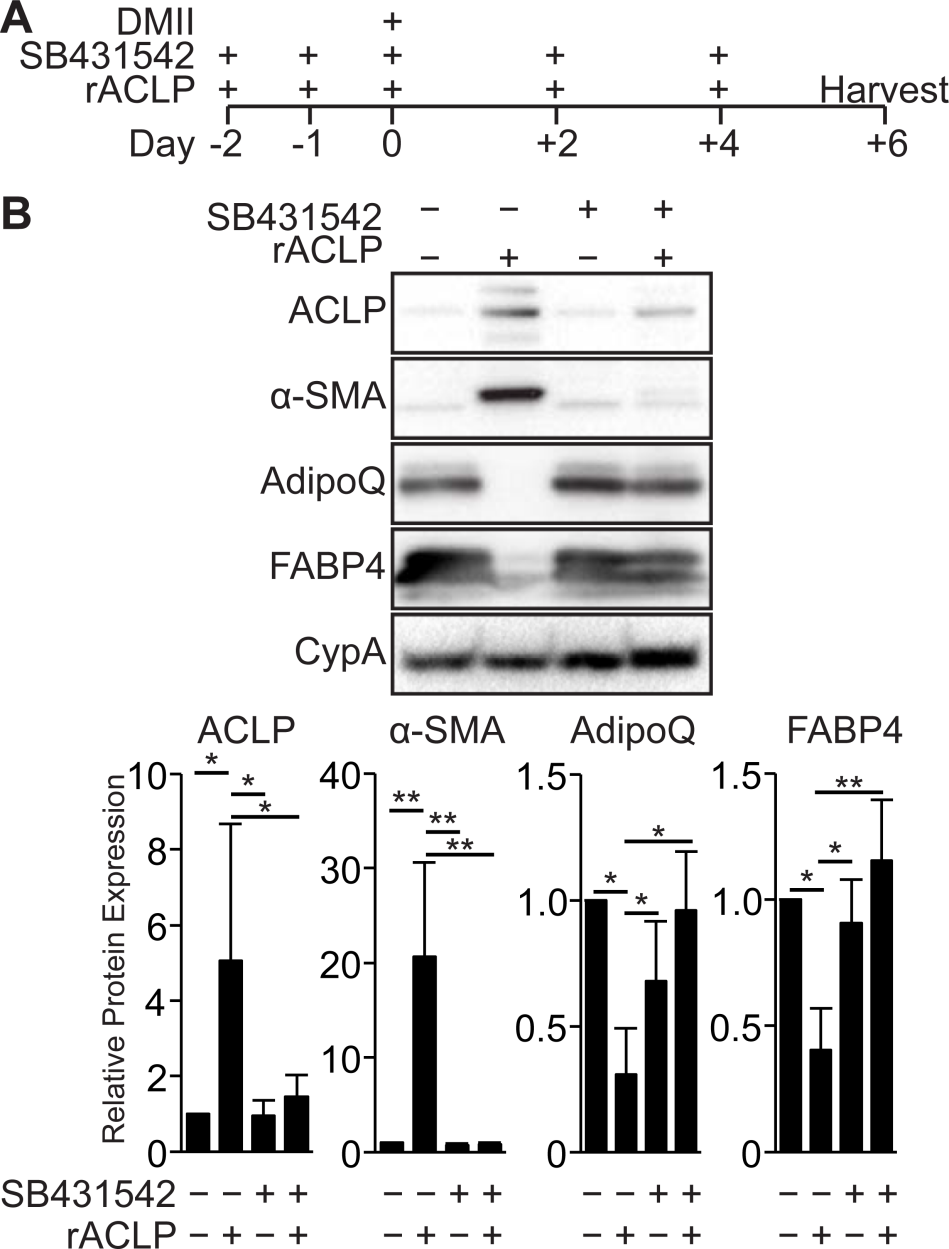
ACLP inhibition of adipogenesis and enhancement of myofibroblast differentiation is dependent on TGFβ receptor activity. **A**, Scheme of 10T1/2 fibroblast adipogenesis with SB431542 and rACLP treatment. **B**, 10T1/2 progenitors were concurrently treated with 30 nM rACLP and vehicle control or 1 μM SB431542, a TGFβ receptor kinase inhibitor, on days -2, -1, 0, +2 and +4 as well as induced to undergo adipogenesis on day 0. Protein was harvested on day +6 and analyzed by SDS-PAGE and Western blot with antibodies against ACLP, α-SMA, AdipoQ, FABP4 and CypA. Protein expression was quantified by densitometry normalized to CypA expression and relatively compared to untreated control cells (n = 3). One way ANOVA with post hoc Tukey’s test for all values (* *p* < 0.05, ** *p* < 0.005). Data presented are expressed as mean ± SD.

Our previous work showed that ACLP signaling exhibited both TGFβ receptor (TGFβR) dependent and independent activity in pulmonary fibroblasts [26]. We investigated whether the effect of ACLP on adipogenesis was dependent on TGFβR signaling pathways. To test this, we treated 10T1/2 fibroblasts with rACLP in the presence or absence of a TGFβR-I kinase inhibitor (SB431542) prior to and throughout adipogenesis (Fig 3A). Compared with untreated controls, rACLP treatment inhibited adipocyte differentiation, indicated by lower expression levels of adiponectin and FABP4 (Fig 3B). Additionally, compared with untreated controls, rACLP enhanced myofibroblast differentiation, as indicated by increased ACLP and α-SMA (Fig 3B). The TGFβR inhibitor alone did not alter expression of ACLP or α-SMA or the ability of cells to undergo adipogenesis. However, addition of the TGFβR inhibitor blocked rACLP-mediated induction of ACLP and α-SMA expression and rescued adipogenesis as indicated by increased FABP4 and adiponectin expression (Fig 3B). These data demonstrate that the ACLP repression of adipogenesis and enhancement of myofibroblast differentiation is dependent on TGFβR activity.

### ACLP accumulation is increased in regions of adipose tissue fibrosis

**Fig 4:**
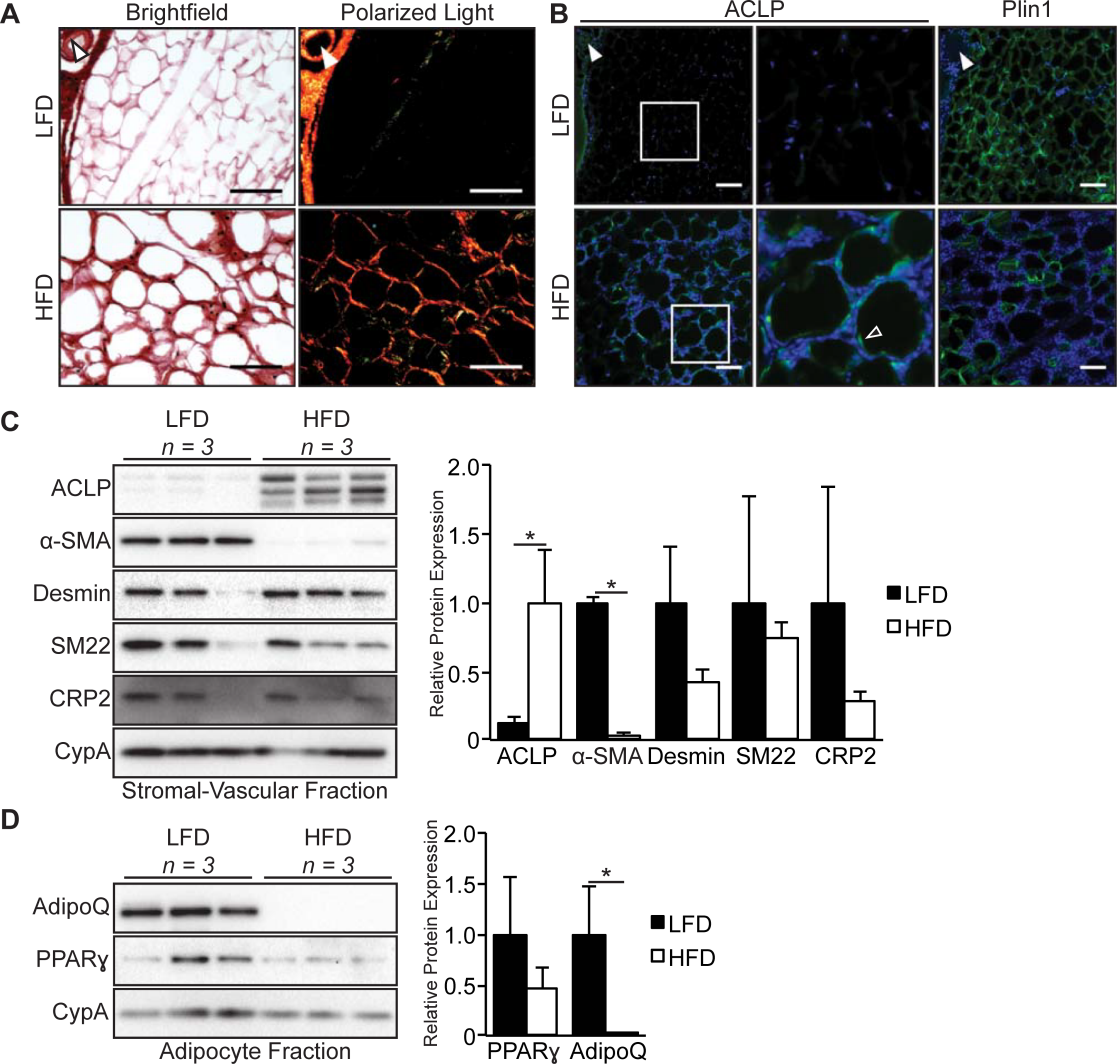
ACLP expression increases in the stromal vascular fraction from diet induced fibrotic epididymal adipose tissue. **A**, epididymal adipose tissue from C57BL6/J male mice on low fat diet or high fat diet for 16 weeks (*n* = 3 for each diet group) was excised and fixed with methyl Carnoy, stained with picrosirius red and imaged using brightfield (left panels) and under polarized light (right panels). **B**, Tissue sections of epididymal adipose tissue following low fat diet or high fat diet were immunostained for ACLP and perilipin and counterstained with DAPI. Data are representative of parallel 10 μm sections. Closed arrowhead indicates vasculature. Open arrowhead indicates peri-cellular staining. Scale bar represents 100 μm. **C**, Epididymal adipose tissue from C57BL6/J male mice on low fat diet or high fat diet for 16 weeks (*n* = 3 for each diet group) was excised, enzymatically digested, fractionated based on buoyancy and then protein lysates were generated of each population. Whole stromal vascular fraction lysate was analyzed by SDS-PAGE and Western blot with antibodies against ACLP, α-SMA, desmin, SM22, CRP2 and CypA. Protein expression was quantified by densitometry normalized to CypA expression and relatively compared to higher expressing diet sample. *, *p* < 0.05 versus low fat diet by Students t-test. **D**, Whole adipocyte lysate was analyzed by SDS-PAGE and Western blot with antibodies against adiponectin, PPARγ and CypA. Protein expression was quantified by densitometry normalized to CypA expression and relatively compared to low fat diet. *, *p* < 0.05 versus low fat diet using Students t-test.

To define the expression of ACLP in fibrotic adipose tissue, we analyzed epididymal white adipose tissue (eWAT) from male mice fed LFD or HFD for 16 weeks. We assessed collagen accumulation by picrosirius red staining and imaging with polarized light [42,43]. In LFD fed mice, collagen was primarily associated with vasculature and was not detected in regions proximal to adipocytes (Fig 4A). In contrast, HFD fed mice exhibited peri-cellular collagen deposition (Fig 4A). This observation was consistent in HFD fed mice, however the extent of peri-cellular collagen deposition varied between mice (S1 Fig). ACLP was strongly associated with the vasculature and not adipocytes in LFD fed animals (Fig 4B, closed arrowhead). ACLP staining co-localized with regions of peri-cellular collagen deposition in adipose tissue isolated from HFD fed mice (Fig 4B, open arrowhead). Consistent with the effects of chronic HFD observed by others [44], perilipin staining was intermittent in these fibrotic regions, indicative of adipocyte cell death (Fig 4B). This observation of perilipin and ACLP staining was consistent in all HFD fed mice in areas of increased nucleus density (S1B Fig). Previous studies have demonstrated that adipocytes secrete numerous ECM proteins in response to diet induced obesity [7]. In order to determine the response and source of ACLP expression to HFD, we performed Western blot analysis on isolated adipocytes and SVF. Following collagenase digestion of LFD and HFD eWAT, we isolated adipocytes and SVF cells based on buoyancy and subsequently generated protein extracts. The SVF of LFD fed male mice expressed significantly less (12% of LFD) ACLP compared to SVF of HFD (Fig 4C). Unexpectedly, α-SMA exhibited the inverse expression pattern with ACLP in the SVF being lower (2% of LFD) in SVF of HFD mice (Fig 4C). To better understand this decrease in α-SMA in the HFD SVF, we examined additional smooth muscle cell markers in these samples. Interestingly, desmin, SM22 and cysteine and glycine rich protein-2 (CRP2) expression was equivalent in SVF of LFD fed and HFD fed mice (Fig 4C). As expected, isolated adipocytes did not express ACLP or α-SMA (not shown). Interestingly, while LFD and HFD derived adipocytes both expressed PPARγ at similar levels, adiponectin was expressed in the LFD fed male mice and its expression was significantly attenuated by HFD (<1% of LFD) (Fig 4D). These findings demonstrate that ACLP expression increases in the SVF in diet-induced fibrotic regions of eWAT.

### Fibrotic adipose tissue SVF cells depleted of inflammatory cells express ACLP

**Fig 5:**
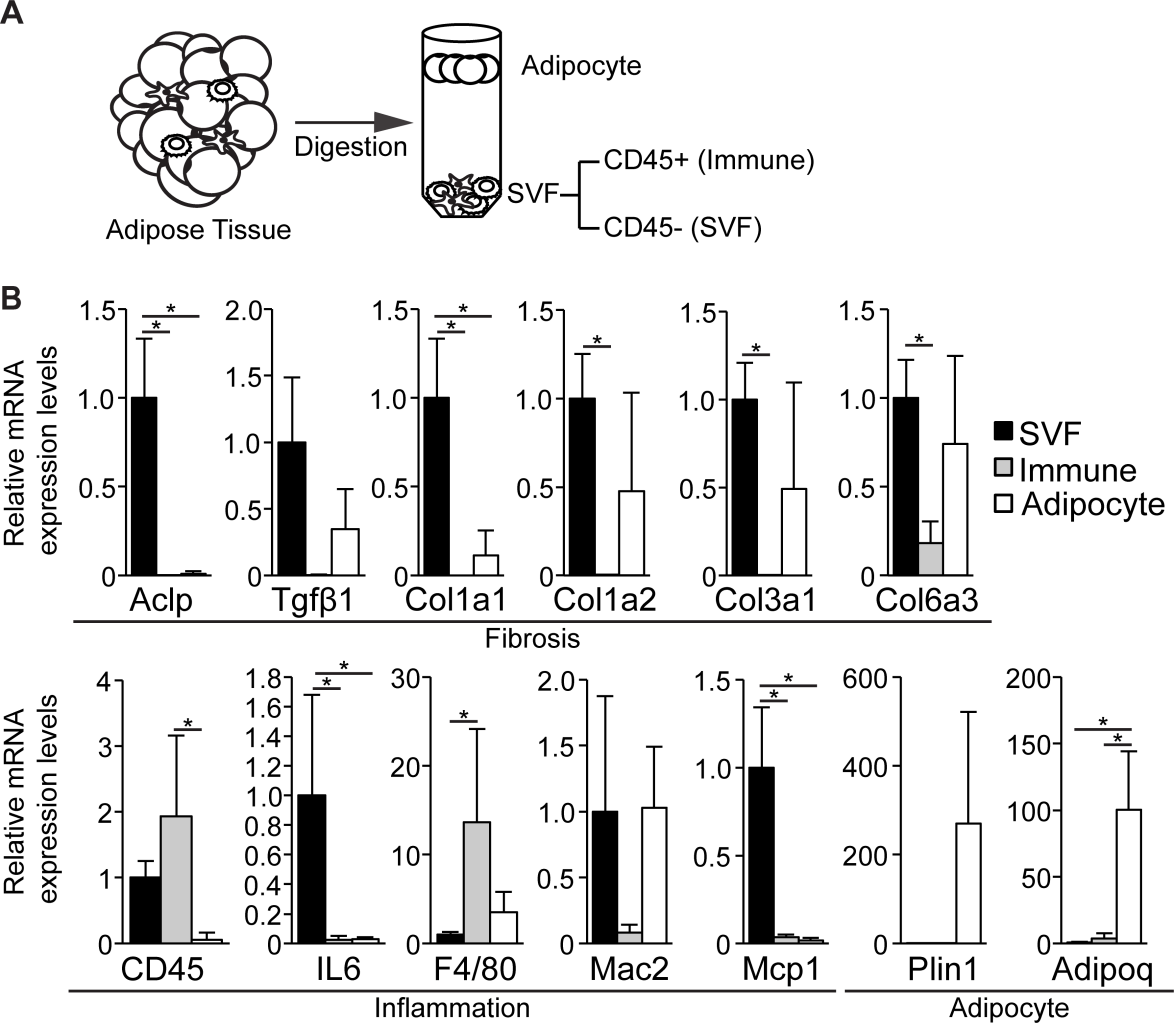
CD45- SVF derived from fibrotic adipose tissue is the source of ACLP expression. **A**, a scheme of isolating SVF CD45-, SVF CD45+ and adipocyte populations from epididymal adipose tissue. **B**, CD45- SVF, CD45+ immune and adipocyte populations were isolated from epididymal adipose tissue from C57BL6/J male mice on high fat diet for 16 weeks (n = 4) and mRNA levels were determined by qPCR analysis of fibrosis, inflammation and adipocyte markers. * *p* < 0.05, one way ANOVA with post hoc Tukey’s test for all values. Data presented are expressed as mean ± SD.

Fibrotic ECM deposition in the adipose tissue correlates with infiltration of immune cells, including macrophages [11,45]. In order to delineate the cellular origin ACLP and collagens in the fibrotic eWAT, we isolated inflammatory and non-inflammatory sub-populations from eWAT of HFD fed male mice. Adipocytes were separated based on buoyancy and the remaining cells were fractionated into CD45+ (immune) and CD45- (SVF) populations (Fig 5A) [46]. Transcript levels of Aclp, Col1a1, IL6 and Mcp1 were higher in SVF cells compared to immune and adipocyte sub-populations. Interestingly, other major ECM components Col1a2, Col3a1 and Col6a3, were similarly expressed in both the SVF and adipocytes and significantly higher compared to immune cells. SVF and immune cells expressed similar amounts of CD45 transcript and higher levels compared to adipocytes. The immune cell fraction expressed significantly higher amounts of, F4/80, compared to SVF, but similar to adipocytes. Adipocytes expressed significantly higher adipoq and higher plin1 compared to SVF and immune cells (Fig 5B). Together these results demonstrate ACLP is produced primarily by stromal-vascular cells, and not by adipocytes or immune cells in the fibrotic eWAT.

### HFD derived SVF cells secrete increased ACLP and have impaired adipogenic potential

**Fig 6:**
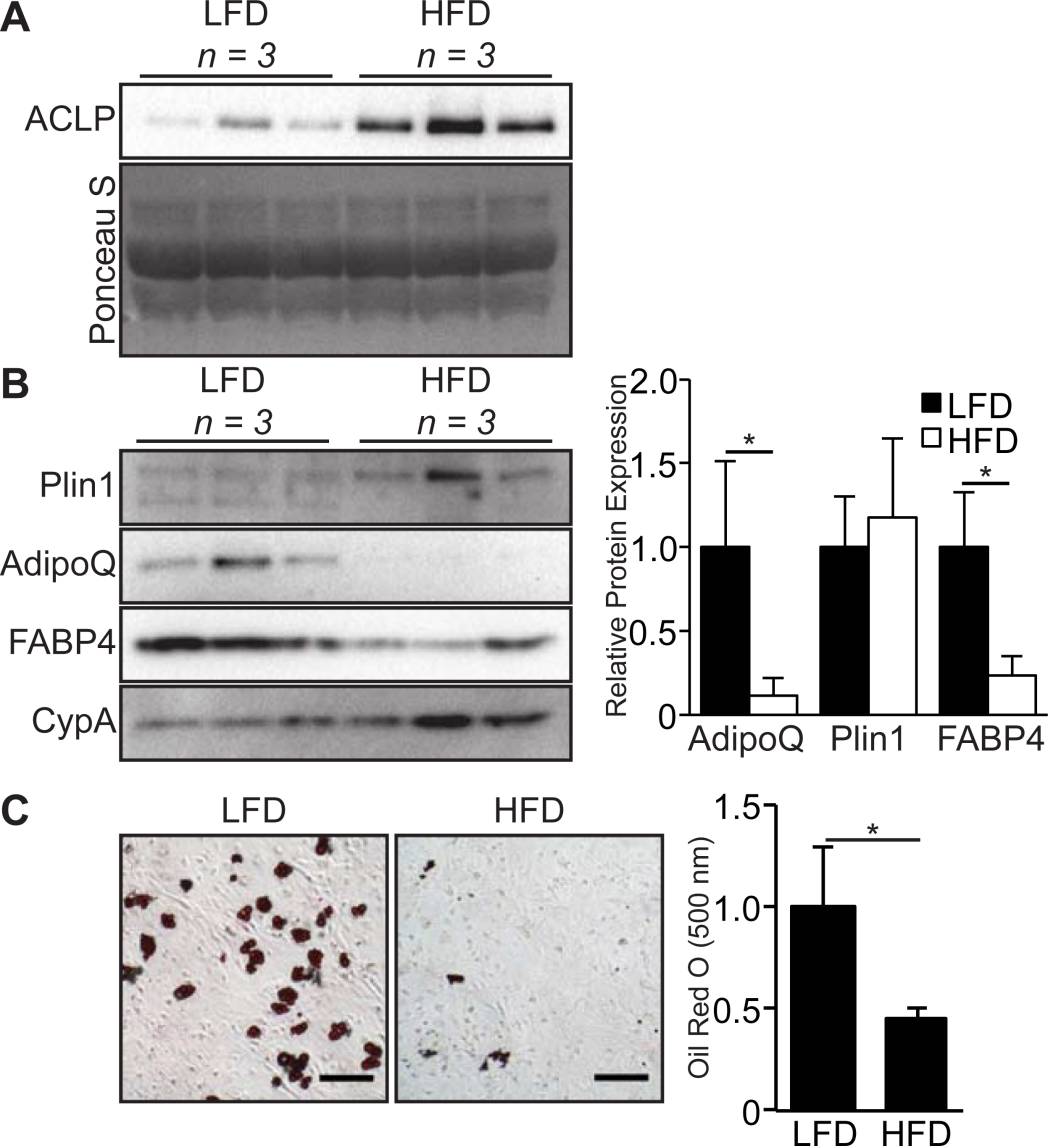
High fat diet reduces adipogenic potential of total SVF. **A**, Epididymal adipose tissue from C57BL6/J male mice on low fat diet or high fat diet for 16 weeks (*n* = 3 for each group) was excised, enzymatically digested and depleted of CD45+ cells. CD45- SVF cells were then cultured for 24 hours in 10% FBS DMEM and subsequently media was collected and analyzed by SDS-PAGE and Western blot with antibodies against ACLP. **B**, CD45- SVF cells were isolated as above and cultured for 10 days in 10% FBS DMEM, DMII was given on day 2 and cell lysates were harvested on day 10. Cell lysates were analyzed by SDS-PAGE and Western blot with antibodies against adiponectin, perilipin and FABP4. Protein expression was quantified by densitometry normalized to CypA expression and relatively compared to low fat diet cells (n = 3). * *p* < 0.05, Students t-test used for all values. **C**, SVF cells were isolated as above and were cultured (100,000 cells/well (12 well)) until confluent, and then induced to undergo adipogenesis with DMII 2 days after confluent. Oil Red O staining and imaging was performed 8 days following adipogenic induction (n = 3). * *p* < 0.05, Students t-test used for value. The scale bar represents 100 μm. Data presented are expressed as mean ± SD.

Recent studies have demonstrated a HFD induces a shift in the adipose progenitor pool towards myofibroblast differentiation [9], however it is unknown how HFD impacts ACLP expression and adipogenic phenotype. As SVF cells are the source of progenitors [30,31,33] and the primary producers of ACLP in the fibrotic eWAT (Fig 5B), we analyzed this SVF sub-population to define the adipogenic phenotype in relation to ACLP secretion. SVF cells depleted of CD45+ cells were purified from LFD and HFD adipose tissue (Fig 5A) and cultured for 24 hours. The media was collected, clarified and analyzed by Western blot for secreted ACLP. Compared with LFD derived SVF, HFD derived SVF secreted increased amounts of ACLP into the media (Fig 6A). HFD derived SVF cells exhibited impaired adipogenesis compared to LFD derived SVF cells, as indicated by significantly lower expression of adiponectin and FABP4 (9.0 and 4.3 fold respectively), however there was similar expression of perilipin (Fig 6B). Consistent with Western blot analysis, we examined lipid droplet accumulation by oil red O staining and compared with controls, HFD derived SVF accumulated 52% less lipid (Fig 6C). These results demonstrate that HFD-induced obesity increases the secretion of ACLP from SVF. Furthermore, the HFD-induced obesity decreases the adipogenic potential of the total SVF. Collectively this supports the inverse relationship of ACLP and adipogenesis.

### ACLP is downregulated during adipogenesis in human adipose stromal cells

**Fig 7:**
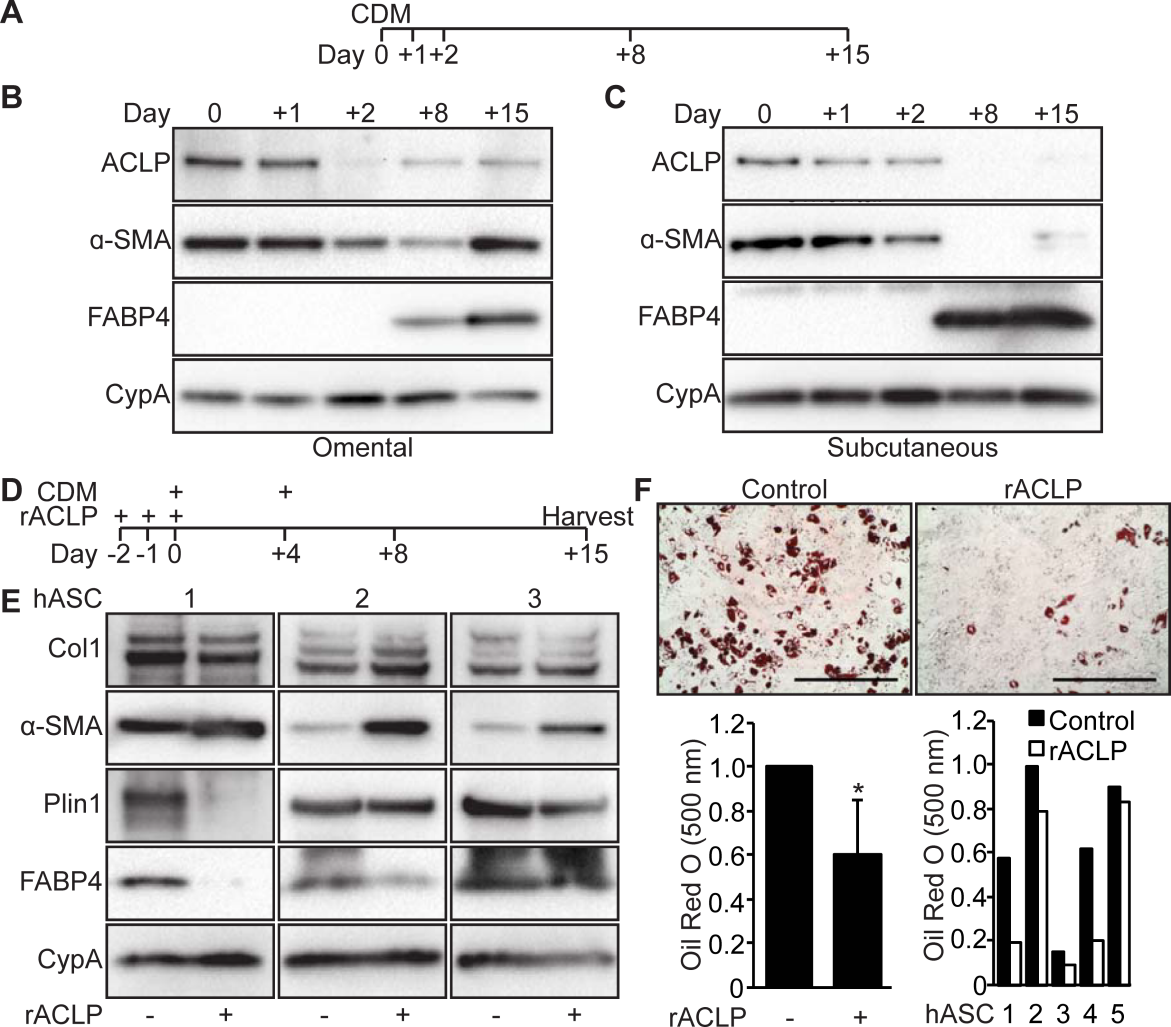
Recombinant ACLP inhibits adipogenesis and enhances myofibroblast differentiation in human adipose stromal cells. **A**, Scheme of time course for adipogenesis of hASC. **B**, hASC derived from subcutaneous adipose depots were induced to undergo adipogenesis with CDM on day 0. Protein was harvested on days 0, +1, +2, +8 and +15. Protein expression was analyzed using SDS-PAGE and Western blot with antibodies against ACLP, α-SMA, FABP4 and cyclophilin-A. **C**, Omental hASC were induced to undergo adipogenesis with CDM on day 0. Protein was harvested on days 0, +1, +2, +8 and +15. Protein expression was analyzed using SDS-PAGE and Western blot with antibodies against ACLP, α-SMA, FABP4 and cyclophilin-A. **D**, Scheme of adipogenesis for hASC and rACLP treatment. **E**, Subcutaneous hASC were treated with 30 nM rACLP on days -2, -1 and 0 and induced to undergo adipogenesis with CDM on day 0 and +4. Protein was harvested on day +15 and analyzed by SDS-PAGE and Western blot with antibodies against collagen, α-SMA, perilipin, FABP4 and CypA (n = 3). **F**, Subcutaneous hASC were treated with 30 nM rACLP on days -2, -1, and 0 and induced to undergo adipogenesis with CDM on day 0 and +4. On day +15 cells were fixed, stained, imaged and quantified with Oil Red O dye (n = 5). Data were normalized relative to control. * *p* < 0.05 versus control. Data presented are expressed as mean ± SD. The scale bar represents 2 mm.

Human adipose tissue depots differentially respond to the stresses of obesity [5,47]. In contrast to the subcutaneous depot, the omental depot is susceptible to fibrotic changes following chronic excess caloric intake [9,48]. Little is currently known about the expression and function of ACLP in human adipogenesis. To determine kinetics of ACLP expression during adipogenesis, primary human adipose stromal cells (hASC) from omental and subcutaneous depots were induced to differentiate with serum-free complete differentiation media (CDM) (Fig 7A) [38]. ACLP and α-SMA protein expression was detected in undifferentiated omental hASC but their expression decreased 2 days following adipogenic induction (Fig 7B). Expression of each re-emerged 8 and 15 days with the concomitant emergence of fatty acid binding protein 4 expression (FABP4) (Fig 7B). A similar early expression pattern of ACLP and α-SMA was observed in differentiating subcutaneous hASC (Fig 7C) but ACLP was only minimally expressed by day 15. Our observations demonstrate that ACLP expression repressed during adipogenesis in omental and subcutaneous human adipose stromal cells.

### Recombinant ACLP represses human adipose stromal cells adipogenesis and promotes myofibroblast differentiation

Omental adipose stromal cells are resistant to adipogenesis compared to subcutaneous adipose stromal cells [49,50]. To test whether rACLP directs the fate of subcutaneous adipose stromal cells towards myofibroblast differentiation, individual subcutaneous hASC isolates were cultured in media containing rACLP (30 nM) prior to adipogenic induction, during the commitment phase [51] (Fig 7D). In comparison to untreated control cells, treated cells expressed lower amounts of perilipin and FABP4 (2/3 and 2/3 cell isolates respectively) and enhanced amounts of α-SMA and Col1 (3/3 and 1/3 cells respectively) at 8 and 15 days following differentiation (Fig 7E). Consistent with an attenuation of adipogenesis, ACLP treated cells also contained 41% less lipid compared to control (Fig 7F). While rACLP treatment can dampen adipogenesis, its effect on subcutaneous hASC varied from donor to donor.

## Discussion

Our studies have uncovered new functions for the secreted protein ACLP in regulating that balance between adipogenic and myofibroblast differentiation fates of progenitors. We report that ACLP expression is downregulated during adipogenesis of mouse precursors and is not primarily in differentiated adipocytes. ACLP signals via the TGFβR to repress adipogenesis and enhance myofibroblast differentiation. ACLP production is enhanced and localized in peri-cellular, collagen rich fibrotic regions of eWAT of HFD fed mice. The principal source of ACLP expression is non-immune stromal vascular cells. Furthermore, our studies have determined that treatment of hASC with rACLP blunted adipogenic differentiation and enhanced myofibroblast differentiation.

TGFβR is a critical mediator of both myofibroblast differentiation and adipogenesis [16–18]. Our studies demonstrate that ACLP enhances myofibroblast differentiation and represses adipogenesis through extracellular activation of the TGFβR (Fig 3B). Additionally, ACLP signaling repressed upregulation of PPARγ protein expression during adipogenesis (Fig 2B) and this likely contributes to the inhibition of adipogenesis. Previously our lab has demonstrated that ACLP enhances TGFβR dependent pathways and its downstream effectors, including SMAD2/3 and myocardin-related transcription factor-A (MRTFA), in differentiating lung fibroblasts [26]. Active TGFβR signaling represses terminal differentiation of adipogenesis by activating SMAD2/3 which complexes with CCAAT/enhancer-binding protein-β and inhibits downstream activation of adipogenic genes [18].

Adipose progenitors are capable of differentiating into multiple cell types, including adipocytes, chondrocytes and osteoblasts [52,53]. Our studies demonstrate that ACLP enhances myofibroblast differentiation and represses adipogenesis (Fig 2B) and is increased in WAT fibrosis (Fig 4). Previous reports have also demonstrated that ACLP expression is increased with idiopathic pulmonary fibrosis and is a critical mediator of myofibroblast differentiation [26,41], together suggesting ACLP is a fibrotic mediator. While our studies demonstrate ACLP enhance myofibroblast differentiation, ACLP may also be involved with enhancement of chondrogenesis and osteogenesis differentiation. Supporting this, ACLP is highly expressed in both connective tissue and skeletal structures in developing mice [34]. Additionally, individuals with an *AEBP1* gene mutation exhibit connective tissue disorders [54]. *In vitro* differentiation of osteoblasts and chondrocytes requires numerous mediators [55], which were not included in these studies. We anticipate that ACLP could potentially participate in chondrogenic and osteogenic differentiation and further studies are required.

It is clear that WAT fibrotic pathways are controlled by inflammation, hypoxia and cell death pathways [4,56], much less is known about the role of ECM associated proteins including ACLP in progenitor differentiation and fibrosis. Our studies support a model where ACLP modulates the differentiation in adipose progenitors from the stromal vascular compartment. ACLP producing cells are non-adipocyte and non-immune cells (Fig 7A and 7B). While CD45 transcripts were expressed by both SVF and immune fractions at comparable levels possibly indicating incomplete fractionation, our results support that the SVF sub-population is the primary producer of ACLP along with other ECM genes. Interestingly, adipose progenitors express ACLP and are also capable of undergoing adipogenesis (Fig 1B) [21,22,36,37]. Together this suggests that adipose progenitors may express but not sufficiently secrete ACLP, or additional mediators may be required to cooperate with ACLP. For instance, our observations of ACLP localization in fibrotic eWAT suggest that ACLP expressing cells expand along with infiltration and expansion of non-ACLP expressing cells (Fig 6B), including immune cells [11,45]. ACLP is a target of TGFβ-TGFβR signaling activity [57,58], which is increased with obesity [19] and obesity induced macrophage infiltration [11]. Together this supports the notion that ACLP secretion and signaling is increased from stromal cells following infiltration of TGFβ secreting immune cells. Our observations of ACLP in fibrotic eWAT demonstrate that ACLP co-localizes among perilipin negative adipocytes (Fig 6B), indicative of cell death which triggers an inflammatory response [44], further supporting that ACLP expression occurs following inflammation. This concept has been demonstrated in experimentally induced lung fibrosis, where genetic ablation of ACLP protected against fibrosis however there was no change in early immune infiltration [41]. Together these data support that ACLP increase in eWAT is a consequence of inflammation and can exacerbate eWAT fibrosis in a non-immune dependent manner.

Several studies have demonstrated the impact of ECM structural properties and composition on adipogenesis and adipose tissue function [7,59,60]. Here we have demonstrated that ACLP is inhibitory to adipogenesis in vitro (Fig 3B and 3C) and localizes with peri-cellular ECM deposition with eWAT fibrosis (Fig 6A and 6B). A protein related to ACLP, carboxypeptidase X-1 (CPX-1) shares structural similarities to ACLP with a central discoidin domains and catalytically inactive metallocarboxypeptidase domain [25], Interestingly, CPX-1 knockdown impairs adipogenesis and results in a reduction of ECM associated proteins [61]. While, ACLP and CPX-1 share structural similarities, they appear to have opposing effects on adipogenesis. While it is unclear the exact impact ACLP has on collagen structure and ECM remodeling, human mutations in *AEBP1* have been recently shown to be causative of connective tissue disorders [54]. Also consistent with a collagen associated function for ACLP, Gusinjac and colleagues observed an anti-adipogenic activity for ACLP in a collagen rich environment [40]. Other studies using proteomics revealed ACLP is increased with fibrotic remodeling in a model of ischemia [58] and abdominal aortic aneurysm [62] which has lead to its designation as a core ECM protein [63].

In numerous tissues, fibrosis is driven by myofibroblasts which are characterized in part by increased co-expression of ECM genes and α-SMA [12]. Our Western blot analysis of male mice eWAT SVF demonstrates a substantial decrease of α-SMA expression in total SVF with HFD (Fig 6C). Others have detected elevated α-SMA expression levels in a subset of SVF cells (PDGFRα+, CD9^high^) with obesity in C3H male mice [9]. The loss of overall α-SMA expression in SVF is potentially due to dedifferentiation of α-SMA+ adipose vascular smooth muscle cells to an α-SMA- ECM producing phenotype, which is well documented in settings of vascular injury [64] and inflammation [65]. Interestingly, the SVF of LFD and HFD expressed comparable levels of other smooth muscle cells markers, SM22, CRP2 and desmin, (Fig 6C). This difference in smooth muscle cell markers and α-SMA suggest either these genes are differentially regulated or the reduction in α-SMA is not related to the vasculature. For instance, in fibrotic eWAT there is a large expansion of cells (Fig 4B) which is likely due to immune cell infiltration [66]. This immune cell infiltration results in potential dilution of α-SMA+ myofibroblasts in the total SVF, thereby reducing the α-SMA expression across the total SVF. Additionally, there is the possibility where the myofibroblast population in fibrotic eWAT are not predominately α-SMA+. While others have demonstrated an increase in α-SMA expression in WAT SVF sub-populations, these may represent a minority of myofibroblasts [5,9,11].

In summary, we demonstrated ACLP enhances the differentiation of mouse and human adipose progenitors to a myofibroblast phenotype at the expense of adipogenic differentiation. Importantly, ACLP is derived from non-adipocytes and non-immune cells, is greatly increased with chronic HFD and localizes with peri-cellular ECM deposition in fibrotic WAT. ACLP activity may represent a novel therapeutic target that is independent of inflammation in ameliorating WAT fibrosis.

## Supporting information

S1 Fig ACLP expression localizes with pericellular ECM deposition. **A**, epididymal adipose tissue from C57BL6/J male mice on low fat diet or high fat diet for 16 weeks (*n* = 3 for each diet group) was excised and fixed with methyl Carnoy, stained with picrosirius red and imaged using brightfield (left panels) and under polarized light (right panels). **B**, Tissue sections of epididymal adipose tissue following low fat diet or high fat diet were immunostained for ACLP and perilipin and counterstained with DAPI. Closed arrowhead indicates vasculature. Open arrowhead indicates peri-cellular staining. Data are from parallel 10 μm sections. Arrow indicates vasculature. Scale bar represents 200 μm.
(EPS)

S1 Table Primer sequences of genes analyzed by qPCR. (EPS)
